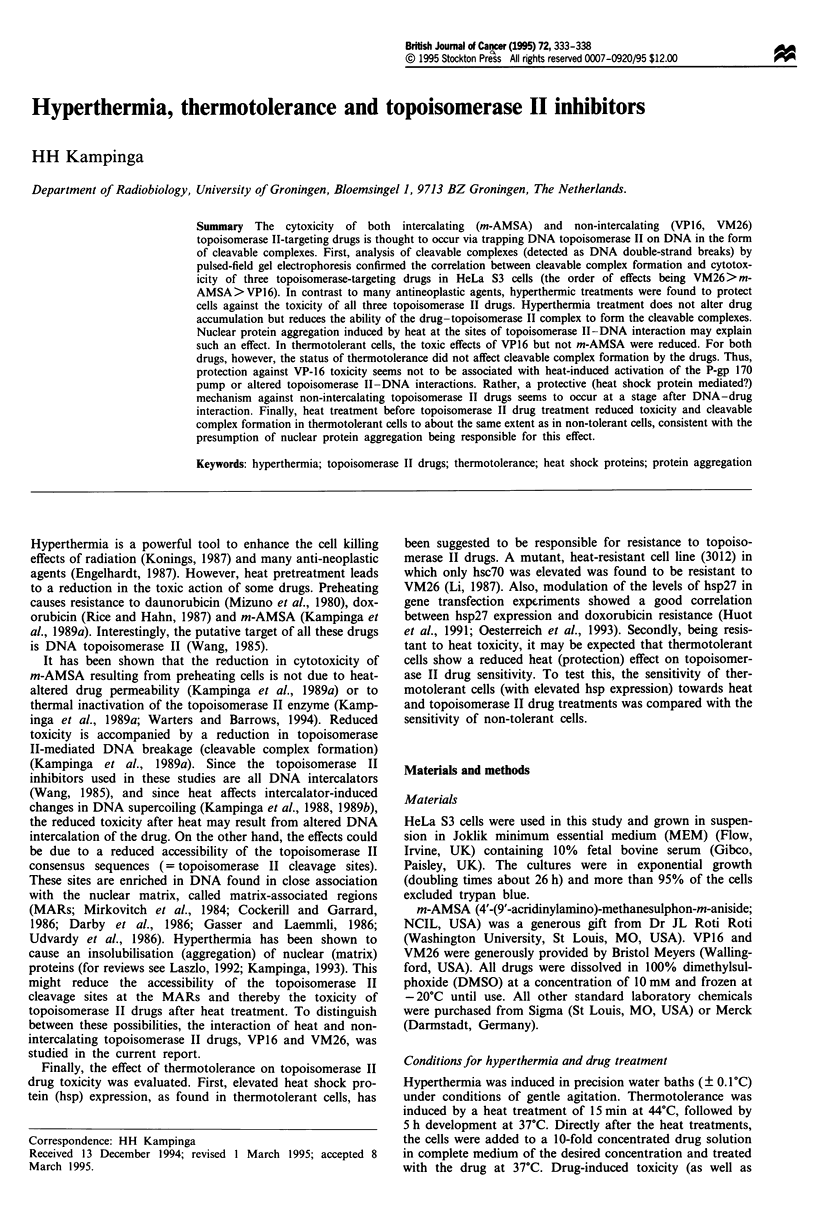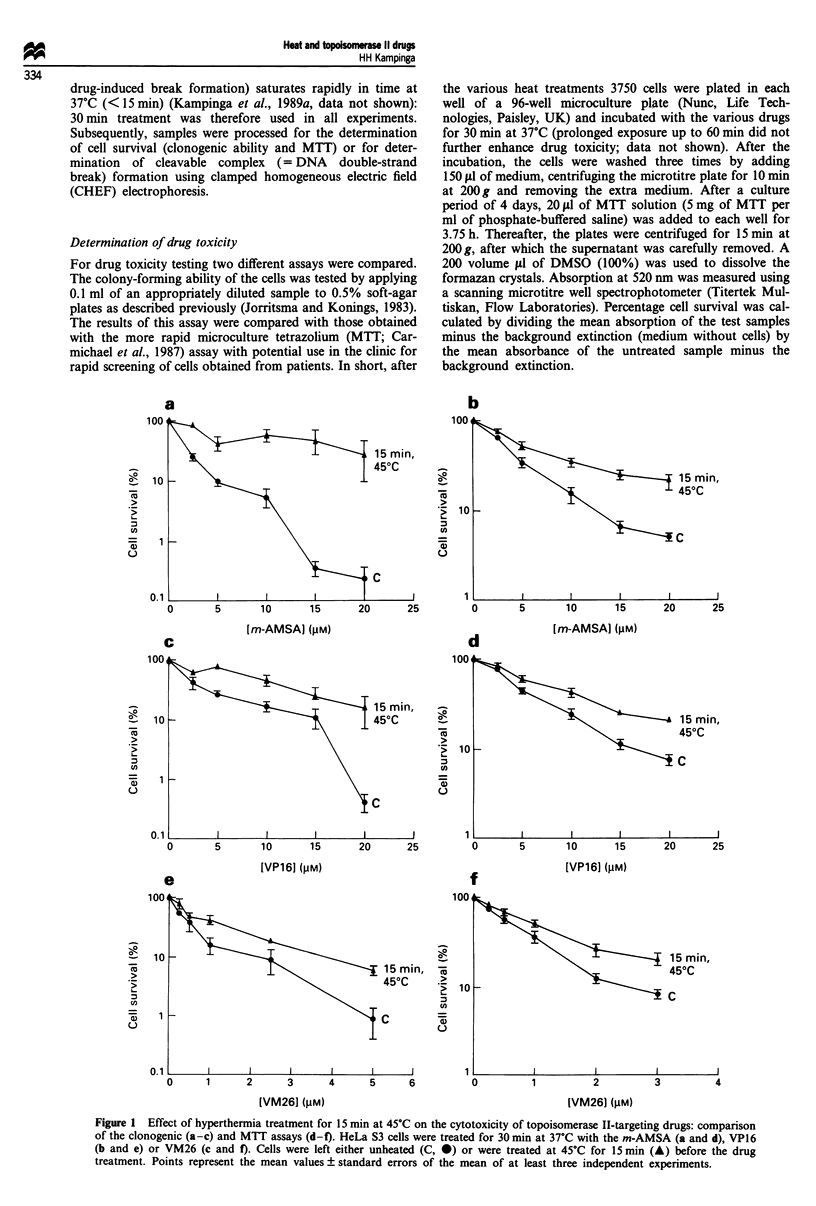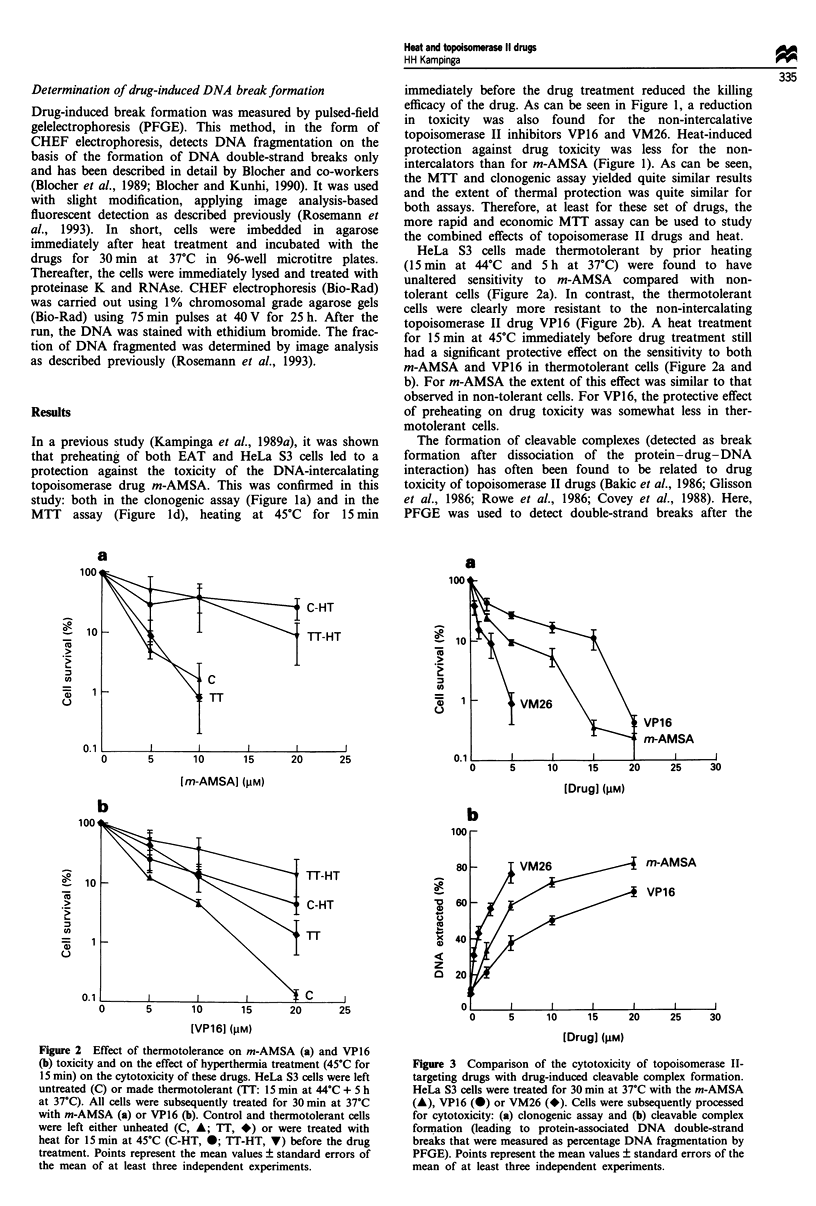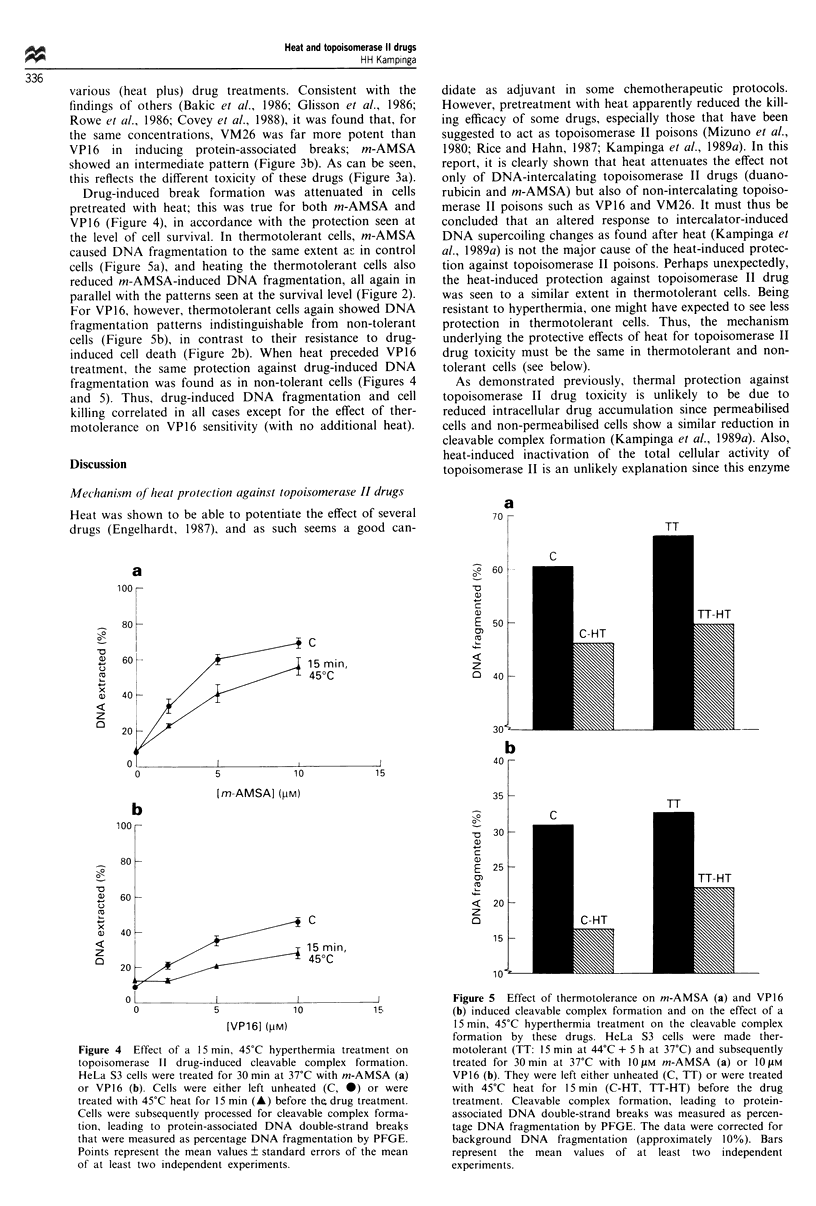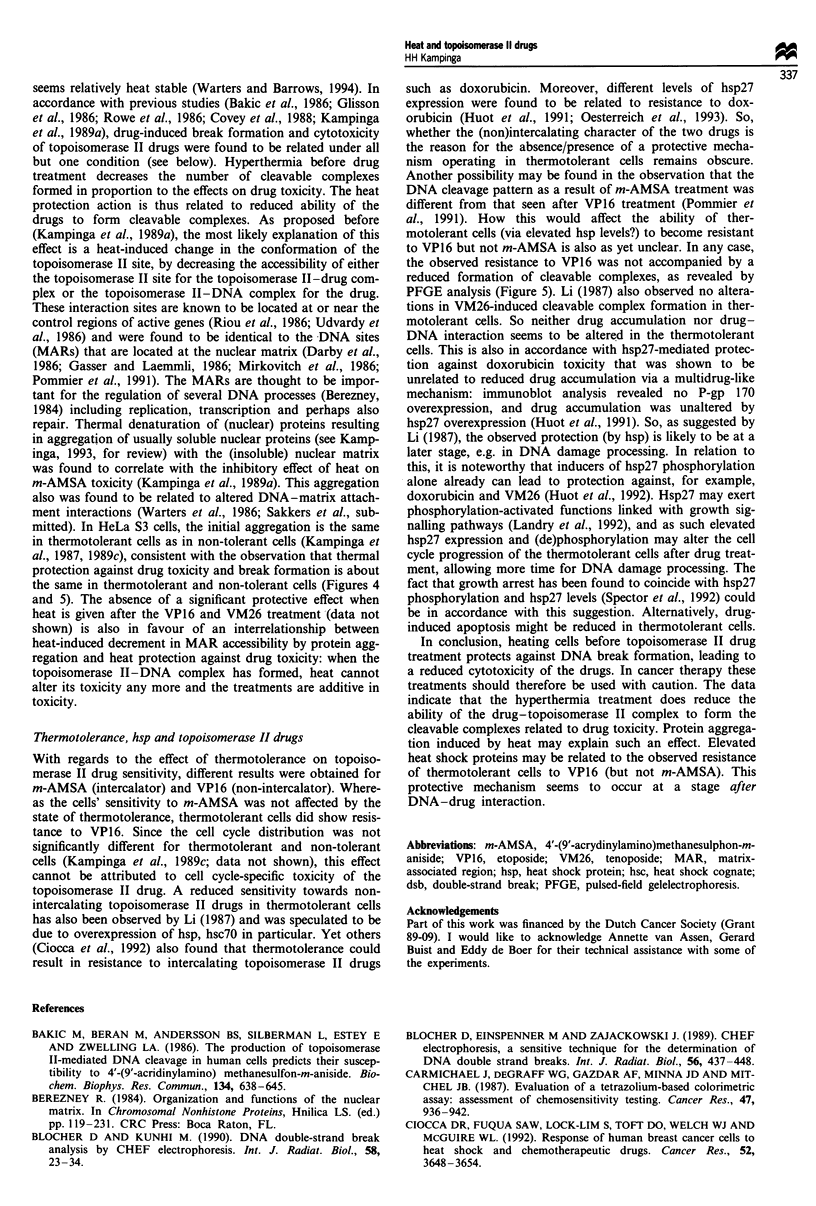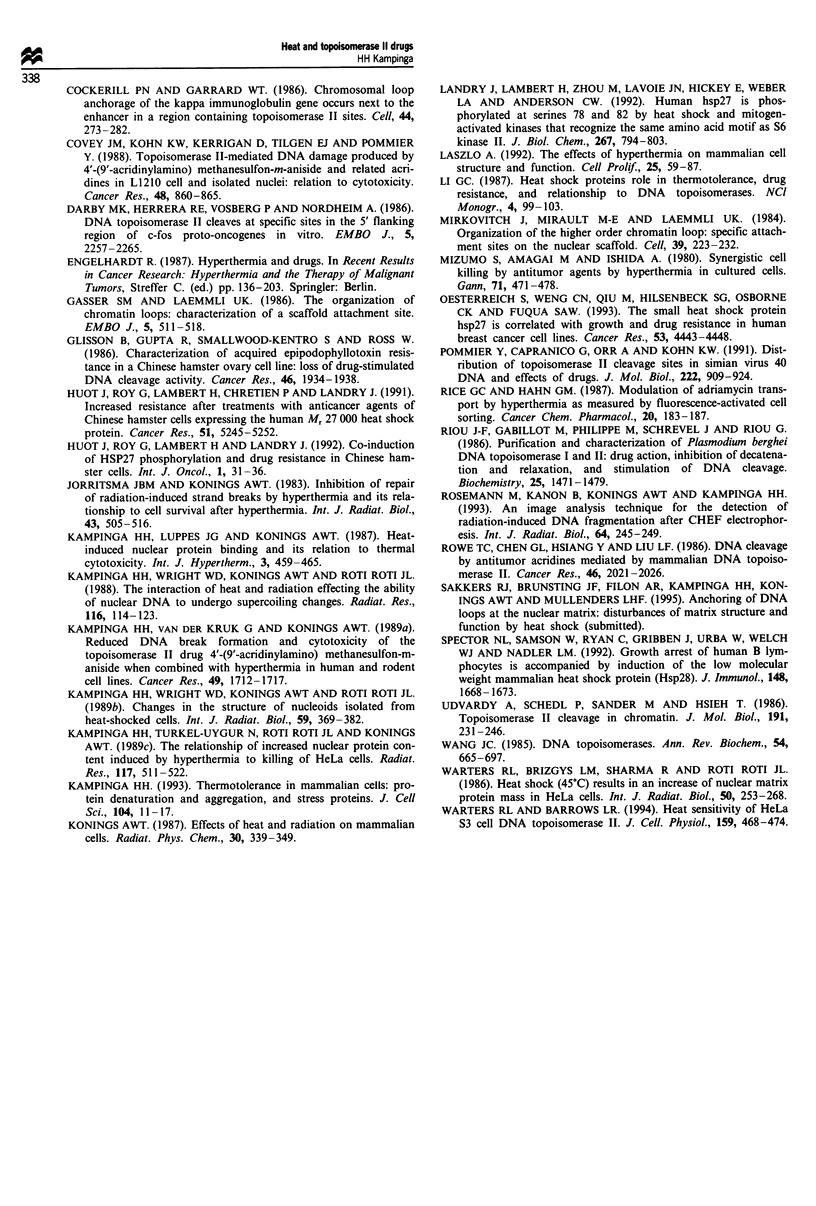# Hyperthermia, thermotolerance and topoisomerase II inhibitors.

**DOI:** 10.1038/bjc.1995.334

**Published:** 1995-08

**Authors:** H. H. Kampinga

**Affiliations:** Department of Radiobiology, University of Groningen, The Netherlands.

## Abstract

The cytoxicity of both intercalating (m-AMSA) and non-intercalating (VP16, VM26) topoisomerase II-targeting drugs is thought to occur via trapping DNA topoisomerase II on DNA in the form of cleavable complexes. First, analysis of cleavable complexes (detected as DNA double-strand breaks) by pulsed-field gel electrophoresis confirmed the correlation between cleavable complex formation and cytotoxicity of three topoisomerase-targeting drugs in HeLa S3 cells (the order of effects being VM26 > m-AMSA > VP16). In contrast to many antineoplastic agents, hyperthermic treatments were found to protect cells against the toxicity of all three topoisomerase II drugs. Hyperthermia treatment does not alter drug accumulation but reduces the ability of the drug-topoisomerase II complex to form the cleavable complexes. Nuclear protein aggregation induced by heat at the sites of topoisomerase II-DNA interaction may explain such an effect. In thermotolerant cells, the toxic effects of VP16 but not m-AMSA were reduced. For both drugs, however, the status of thermotolerance did not affect cleavable complex formation by the drugs. Thus, protection against VP-16 toxicity seems not to be associated with heat-induced activation of the P-gp 170 pump or altered topoisomerase II-DNA interactions. Rather, a protective (heat shock protein mediated?) mechanism against non-intercalating topoisomerase II drugs seems to occur at a stage after DNA-drug interaction. Finally, heat treatment before topoisomerase II drug treatment reduced toxicity and cleavable complex formation in thermotolerant cells to about the same extent as in non-tolerant cells, consistent with the presumption of nuclear protein aggregation being responsible for this effect.


British Journal of Cancer (1995) 72, 333–338
© 1995 Stockton Press All rights reserved 0007–0920/95 $12.00

### Hyperthermia, thermotolerance and topoisomerase II inhibitors

HH Kampinga

*Department of Radiobiology, University of Groningen, Bloemsingel 1, 9713 BZ Groningen, The Netherlands.*

**Summary** The cytoxicity of both intercalating (*m*-AMSA) and non-intercalating (VP16, VM26) topoisomerase II-targeting drugs is thought to occur via trapping DNA topoisomerase II on DNA in the form of cleavable complexes. First, analysis of cleavable complexes (detected as DNA double-strand breaks) by pulsed-field gel electrophoresis confirmed the correlation between cleavable complex formation and cytotoxicity of three topoisomerase-targeting drugs in HeLa S3 cells (the order of effects being VM26 > *m*-AMSA > VP16). In contrast to many antineoplastic agents, hyperthermic treatments were found to protect cells against the toxicity of all three topoisomerase II drugs. Hyperthermia treatment does not alter drug accumulation but reduces the ability of the drug–topoisomerase II complex to form the cleavable complexes. Nuclear protein aggregation induced by heat at the sites of topoisomerase II–DNA interaction may explain such an effect. In thermotolerant cells, the toxic effects of VP16 but not *m*-AMSA were reduced. For both drugs, however, the status of thermotolerance did not affect cleavable complex formation by the drugs. Thus, protection against VP-16 toxicity seems not to be associated with heat-induced activation of the P-gp 170 pump or altered topoisomerase II–DNA interactions. Rather, a protective (heat shock protein mediated?) mechanism against non-intercalating topoisomerase II drugs seems to occur at a stage after DNA–drug interaction. Finally, heat treatment before topoisomerase II drug treatment reduced toxicity and cleavable complex formation in thermotolerant cells to about the same extent as in non-tolerant cells, consistent with the presumption of nuclear protein aggregation being responsible for this effect.

**Keywords**: hyperthermia; topoisomerase II drugs; thermotolerance; heat shock proteins; protein aggregation

Hyperthermia is a powerful tool to enhance the cell killing effects of radiation (Konings, 1987) and many anti-neoplastic agents (Engelhardt, 1987). However, heat pretreatment leads to a reduction in the toxic action of some drugs. Preheating causes resistance to daunorubicin (Mizuno *et al.*, 1980), doxorubicin (Rice and Hahn, 1987) and *m*-AMSA (Kampinga *et al.*, 1989*a*). Interestingly, the putative target of all these drugs is DNA topoisomerase II (Wang, 1985).

It has been shown that the reduction in cytotoxicity of *m*-AMSA resulting from preheating cells is not due to heat-altered drug permeability (Kampinga *et al.*, 1989*a*) or to thermal inactivation of the topoisomerase II enzyme (Kampinga *et al.*, 1989*a*; Warters and Barrows, 1994). Reduced toxicity is accompanied by a reduction in topoisomerase II-mediated DNA breakage (cleavable complex formation) (Kampinga *et al.*, 1989*a*). Since the topoisomerase II inhibitors used in these studies are all DNA intercalators (Wang, 1985), and since heat affects intercalator-induced changes in DNA supercoiling (Kampinga *et al.*, 1988, 1989*b*), the reduced toxicity after heat may result from altered DNA intercalation of the drug. On the other hand, the effects could be due to a reduced accessibility of the topoisomerase II consensus sequences (= topoisomerase II cleavage sites). These sites are enriched in DNA found in close association with the nuclear matrix, called matrix-associated regions (MARs; Mirkovitch *et al.*, 1984; Cockerill and Garrard, 1986; Darby *et al.*, 1986; Gasser and Laemmli, 1986; Udvardy *et al.*, 1986). Hyperthermia has been shown to cause an insolubilisation (aggregation) of nuclear (matrix) proteins (for reviews see Laszlo, 1992; Kampinga, 1993). This might reduce the accessibility of the topoisomerase II cleavage sites at the MARs and thereby the toxicity of topoisomerase II drugs after heat treatment. To distinguish between these possibilities, the interaction of heat and non-intercalating topoisomerase II drugs, VP16 and VM26, was studied in the current report.

Finally, the effect of thermotolerance on topoisomerase II drug toxicity was evaluated. First, elevated heat shock protein (hsp) expression, as found in thermotolerant cells, has been suggested to be responsible for resistance to topoisomerase II drugs. A mutant, heat-resistant cell line (3012) in which only hsc70 was elevated was found to be resistant to VM26 (Li, 1987). Also, modulation of the levels of hsp27 in gene transfection experiments showed a good correlation between hsp27 expression and doxorubicin resistance (Huot *et al.*, 1991; Oesterreich *et al.*, 1993). Secondly, being resistant to heat toxicity, it may be expected that thermotolerant cells show a reduced heat (protection) effect on topoisomerase II drug sensitivity. To test this, the sensitivity of thermotolerant cells (with elevated hsp expression) towards heat and topoisomerase II drug treatments was compared with the sensitivity of non-tolerant cells.

### Materials and methods

### *Materials*

HeLa S3 cells were used in this study and grown in suspension in Joklik minimum essential medium (MEM) (Flow, Irvine, UK) containing 10% fetal bovine serum (Gibco, Paisley, UK). The cultures were in exponential growth (doubling times about 26 h) and more than 95% of the cells excluded trypan blue.

*m*-AMSA (4′-(9′-acridinylamino)-methanesulphon-*m*-aniside; NCIL, USA) was a generous gift from Dr JL Roti Roti (Washington University, St Louis, MO, USA). VP16 and VM26 were generously provided by Bristol Meyers (Wallingford, USA). All drugs were dissolved in 100% dimethylsulphoxide (DMSO) at a concentration of 10 mM and frozen at −20°C until use. All other standard laboratory chemicals were purchased from Sigma (St Louis, MO, USA) or Merck (Darmstadt, Germany).

### *Conditions for hyperthermia and drug treatment*

Hyperthermia was induced in precision water baths (± 0.1°C) under conditions of gentle agitation. Thermotolerance was induced by a heat treatment of 15 min at 44°C, followed by 5 h development at 37°C. Directly after the heat treatments, the cells were added to a 10-fold concentrated drug solution in complete medium of the desired concentration and treated with the drug at 37°C. Drug-induced toxicity (as well as

Correspondence: HH Kampinga
Received 13 December 1994; revised 1 March 1995; accepted 8 March 1995.

drug-induced break formation) saturates rapidly in time at 37°C (<15 min) (Kampinga *et al.*, 1989*a*, data not shown): 30 min treatment was therefore used in all experiments. Subsequently, samples were processed for the determination of cell survival (clonogenic ability and MTT) or for determination of cleavable complex (= DNA double-strand break) formation using clamped homogeneous electric field (CHEF) electrophoresis.

### *Determination of drug toxicity*

For drug toxicity testing two different assays were compared. The colony-forming ability of the cells was tested by applying 0.1 ml of an appropriately diluted sample to 0.5% soft-agar plates as described previously (Jorritsma and Konings, 1983). The results of this assay were compared with those obtained with the more rapid microculture tetrazolium (MTT; Carmichael *et al.*, 1987) assay with potential use in the clinic for rapid screening of cells obtained from patients. In short, after the various heat treatments 3750 cells were plated in each well of a 96-well microculture plate (Nunc, Life Technologies, Paisley, UK) and incubated with the various drugs for 30 min at 37°C (prolonged exposure up to 60 min did not further enhance drug toxicity; data not shown). After the incubation, the cells were washed three times by adding 150 μl of medium, centrifuging the microtitre plate for 10 min at 200 *g* and removing the extra medium. After a culture period of 4 days, 20 μl of MTT solution (5 mg of MTT per ml of phosphate-buffered saline) was added to each well for 3.75 h. Thereafter, the plates were centrifuged for 15 min at 200 *g*, after which the supernatant was carefully removed. A 200 volume μl of DMSO (100%) was used to dissolve the formazan crystals. Absorption at 520 nm was measured using a scanning microtitre well spectrophotometer (Titertek Multiskan, Flow Laboratories). Percentage cell survival was calculated by dividing the mean absorption of the test samples minus the background extinction (medium without cells) by the mean absorbance of the untreated sample minus the background extinction.

**Figure 1** Effect of hyperthermia treatment for 15 min at 45°C on the cytotoxicity of topoisomerase II-targeting drugs: comparison of the clonogenic (**a**–**c**) and MTT assays (**d**–**f**). HeLa S3 cells were treated for 30 min at 37°C with the *m*-AMSA (**a** and **d**), VP16 (**b** and **e**) or VM26 (**c** and **f**). Cells were left either unheated (C, ●) or were treated at 45°C for 15 min (▲) before the drug treatment. Points represent the mean values ± standard errors of the mean of at least three independent experiments.

### Determination of drug-induced DNA break formation

Drug-induced break formation was measured by pulsed-field gelelectrophoresis (PFGE). This method, in the form of CHEF electrophoresis, detects DNA fragmentation on the basis of the formation of DNA double-strand breaks only and has been described in detail by Blocher and co-workers (Blocher *et al.*, 1989; Blocher and Kunhi, 1990). It was used with slight modification, applying image analysis-based fluorescent detection as described previously (Rosemann *et al.*, 1993). In short, cells were imbedded in agarose immediately after heat treatment and incubated with the drugs for 30 min at 37°C in 96-well microtitre plates. Thereafter, the cells were immediately lysed and treated with proteinase K and RNAse. CHEF electrophoresis (Bio-Rad) was carried out using 1% chromosomal grade agarose gels (Bio-Rad) using 75 min pulses at 40 V for 25 h. After the run, the DNA was stained with ethidium bromide. The fraction of DNA fragmented was determined by image analysis as described previously (Rosemann *et al.*, 1993).

### Results

In a previous study (Kampinga *et al.*, 1989*a*), it was shown that preheating of both EAT and HeLa S3 cells led to a protection against the toxicity of the DNA-intercalating topoisomerase drug *m*-AMSA. This was confirmed in this study: both in the clonogenic assay (Figure 1a) and in the MTT assay (Figure 1d), heating at 45°C for 15 min immediately before the drug treatment reduced the killing efficacy of the drug. As can be seen in Figure 1, a reduction in toxicity was also found for the non-intercalative topoisomerase II inhibitors VP16 and VM26. Heat-induced protection against drug toxicity was less for the non-intercalators than for *m*-AMSA (Figure 1). As can be seen, the MTT and clonogenic assay yielded quite similar results and the extent of thermal protection was quite similar for both assays. Therefore, at least for these set of drugs, the more rapid and economic MTT assay can be used to study the combined effects of topoisomerase II drugs and heat.

HeLa S3 cells made thermotolerant by prior heating (15 min at 44°C and 5 h at 37°C) were found to have unaltered sensitivity to *m*-AMSA compared with non-tolerant cells (Figure 2a). In contrast, the thermotolerant cells were clearly more resistant to the non-intercalating topoisomerase II drug VP16 (Figure 2b). A heat treatment for 15 min at 45°C immediately before drug treatment still had a significant protective effect on the sensitivity to both *m*-AMSA and VP16 in thermotolerant cells (Figure 2a and b). For *m*-AMSA the extent of this effect was similar to that observed in non-tolerant cells. For VP16, the protective effect of preheating on drug toxicity was somewhat less in thermotolerant cells.

The formation of cleavable complexes (detected as break formation after dissociation of the protein–drug–DNA interaction) has often been found to be related to drug toxicity of topoisomerase II drugs (Bakic *et al.*, 1986; Glisson *et al.*, 1986; Rowe *et al.*, 1986; Covey *et al.*, 1988). Here, PFGE was used to detect double-strand breaks after the

**Figure 2** Effect of thermotolerance on *m*-AMSA (**a**) and VP16 (**b**) toxicity and on the effect of hyperthermia treatment (45°C for 15 min) on the cytotoxicity of these drugs. HeLa S3 cells were left untreated (C) or made thermotolerant (TT: 15 min at 44°C + 5 h at 37°C). All cells were subsequently treated for 30 min at 37°C with *m*-AMSA (**a**) or VP16 (**b**). Control and thermotolerant cells were left either unheated (C, ▲; TT, ◆) or were treated with heat for 15 min at 45°C (C-HT, ●; TT-HT, ▼) before the drug treatment. Points represent the mean values ± standard errors of the mean of at least three independent experiments.

**Figure 3** Comparison of the cytotoxicity of topoisomerase II-targeting drugs with drug-induced cleavable complex formation. HeLa S3 cells were treated for 30 min at 37°C with the *m*-AMSA (▲), VP16 (●) or VM26 (◆). Cells were subsequently processed for cytotoxicity: (**a**) clonogenic assay and (**b**) cleavable complex formation (leading to protein-associated DNA double-strand breaks that were measured as percentage DNA fragmentation by PFGE). Points represent the mean values ± standard errors of the mean of at least three independent experiments.

various (heat plus) drug treatments. Consistent with the findings of others (Bakic *et al.*, 1986; Glisson *et al.*, 1986; Rowe *et al.*, 1986; Covey *et al.*, 1988), it was found that, for the same concentrations, VM26 was far more potent than VP16 in inducing protein-associated breaks; *m*-AMSA showed an intermediate pattern (Figure 3b). As can be seen, this reflects the different toxicity of these drugs (Figure 3a).

Drug-induced break formation was attenuated in cells pretreated with heat; this was true for both *m*-AMSA and VP16 (Figure 4), in accordance with the protection seen at the level of cell survival. In thermotolerant cells, *m*-AMSA caused DNA fragmentation to the same extent as in control cells (Figure 5a), and heating the thermotolerant cells also reduced *m*-AMSA-induced DNA fragmentation, all again in parallel with the patterns seen at the survival level (Figure 2). For VP16, however, thermotolerant cells again showed DNA fragmentation patterns indistinguishable from non-tolerant cells (Figure 5b), in contrast to their resistance to drug-induced cell death (Figure 2b). When heat preceded VP16 treatment, the same protection against drug-induced DNA fragmentation was found as in non-tolerant cells (Figures 4 and 5). Thus, drug-induced DNA fragmentation and cell killing correlated in all cases except for the effect of thermotolerance on VP16 sensitivity (with no additional heat).

### Discussion

*Mechanism of heat protection against topoisomerase II drugs*

Heat was shown to be able to potentiate the effect of several drugs (Engelhardt, 1987), and as such seems a good candidate as adjuvant in some chemotherapeutic protocols. However, pretreatment with heat apparently reduced the killing efficacy of some drugs, especially those that have been suggested to act as topoisomerase II poisons (Mizuno *et al.*, 1980; Rice and Hahn, 1987; Kampinga *et al.*, 1989*a*). In this report, it is clearly shown that heat attenuates the effect not only of DNA-intercalating topoisomerase II drugs (duanorubicin and *m*-AMSA) but also of non-intercalating topoisomerase II poisons such as VP16 and VM26. It must thus be concluded that an altered response to intercalator-induced DNA supercoiling changes as found after heat (Kampinga *et al.*, 1989*a*) is not the major cause of the heat-induced protection against topoisomerase II poisons. Perhaps unexpectedly, the heat-induced protection against topoisomerase II drug was seen to a similar extent in thermotolerant cells. Being resistant to hyperthermia, one might have expected to see less protection in thermotolerant cells. Thus, the mechanism underlying the protective effects of heat for topoisomerase II drug toxicity must be the same in thermotolerant and non-tolerant cells (see below).

As demonstrated previously, thermal protection against topoisomerase II drug toxicity is unlikely to be due to reduced intracellular drug accumulation since permeabilised cells and non-permeabilised cells show a similar reduction in cleavable complex formation (Kampinga *et al.*, 1989*a*). Also, heat-induced inactivation of the total cellular activity of topoisomerase II is an unlikely explanation since this enzyme

**Figure 4** Effect of a 15 min, 45°C hyperthermia treatment on topoisomerase II drug-induced cleavable complex formation. HeLa S3 cells were treated for 30 min at 37°C with *m*-AMSA (**a**) or VP16 (**b**). Cells were either left unheated (C, ●) or were treated with 45°C heat for 15 min (▲) before the drug treatment. Cells were subsequently processed for cleavable complex formation, leading to protein-associated DNA double-strand breaks that were measured as percentage DNA fragmentation by PFGE. Points represent the mean values ± standard errors of the mean of at least two independent experiments.

**Figure 5** Effect of thermotolerance on *m*-AMSA (**a**) and VP16 (**b**) induced cleavable complex formation and on the effect of a 15 min, 45°C hyperthermia treatment on the cleavable complex formation by these drugs. HeLa S3 cells were made thermotolerant (TT: 15 min at 44°C + 5 h at 37°C) and subsequently treated for 30 min at 37°C with 10 μM *m*-AMSA (**a**) or 10 μM VP16 (**b**). They were left either unheated (C, TT) or were treated with 45°C heat for 15 min (C-HT, TT-HT) before the drug treatment. Cleavable complex formation, leading to protein-associated DNA double-strand breaks was measured as percentage DNA fragmentation by PFGE. The data were corrected for background DNA fragmentation (approximately 10%). Bars represent the mean values of at least two independent experiments.

seems relatively heat stable (Warters and Barrows, 1994). In accordance with previous studies (Bakic *et al.*, 1986; Glisson *et al.*, 1986; Rowe *et al.*, 1986; Covey *et al.*, 1988; Kampinga *et al.*, 1989*a*), drug-induced break formation and cytotoxicity of topoisomerase II drugs were found to be related under all but one condition (see below). Hyperthermia before drug treatment decreases the number of cleavable complexes formed in proportion to the effects on drug toxicity. The heat protection action is thus related to reduced ability of the drugs to form cleavable complexes. As proposed before (Kampinga *et al.*, 1989*a*), the most likely explanation of this effect is a heat-induced change in the conformation of the topoisomerase II site, by decreasing the accessibility of either the topoisomerase II site for the topoisomerase II-drug complex or the topoisomerase II-DNA complex for the drug. These interaction sites are known to be located at or near the control regions of active genes (Riou *et al.*, 1986; Udvardy *et al.*, 1986) and were found to be identical to the DNA sites (MARs) that are located at the nuclear matrix (Darby *et al.*, 1986; Gasser and Laemmli, 1986; Mirkovitch *et al.*, 1986; Pommier *et al.*, 1991). The MARs are thought to be important for the regulation of several DNA processes (Berezney, 1984) including replication, transcription and perhaps also repair. Thermal denaturation of (nuclear) proteins resulting in aggregation of usually soluble nuclear proteins (see Kampinga, 1993, for review) with the (insoluble) nuclear matrix was found to correlate with the inhibitory effect of heat on *m*-AMSA toxicity (Kampinga *et al.*, 1989*a*). This aggregation also was found to be related to altered DNA-matrix attachment interactions (Warters *et al.*, 1986; Sakkers *et al.*, submitted). In HeLa S3 cells, the initial aggregation is the same in thermotolerant cells as in non-tolerant cells (Kampinga *et al.*, 1987, 1989*c*), consistent with the observation that thermal protection against drug toxicity and break formation is about the same in thermotolerant and non-tolerant cells (Figures 4 and 5). The absence of a significant protective effect when heat is given after the VP16 and VM26 treatment (data not shown) is also in favour of an interrelationship between heat-induced decrement in MAR accessibility by protein aggregation and heat protection against drug toxicity: when the topoisomerase II-DNA complex has formed, heat cannot alter its toxicity any more and the treatments are additive in toxicity.

### *Thermotolerance, hsp and topoisomerase II drugs*

With regards to the effect of thermotolerance on topoisomerase II drug sensitivity, different results were obtained for *m*-AMSA (intercalator) and VP16 (non-intercalator). Whereas the cells' sensitivity to *m*-AMSA was not affected by the state of thermotolerance, thermotolerant cells did show resistance to VP16. Since the cell cycle distribution was not significantly different for thermotolerant and non-tolerant cells (Kampinga *et al.*, 1989*c*; data not shown), this effect cannot be attributed to cell cycle-specific toxicity of the topoisomerase II drug. A reduced sensitivity towards non-intercalating topoisomerase II drugs in thermotolerant cells has also been observed by Li (1987) and was speculated to be due to overexpression of hsp, hsc70 in particular. Yet others (Ciocca *et al.*, 1992) also found that thermotolerance could result in resistance to intercalating topoisomerase II drugs such as doxorubicin. Moreover, different levels of hsp27 expression were found to be related to resistance to doxorubicin (Huot *et al.*, 1991; Oesterreich *et al.*, 1993). So, whether the (non)intercalating character of the two drugs is the reason for the absence/presence of a protective mechanism operating in thermotolerant cells remains obscure. Another possibility may be found in the observation that the DNA cleavage pattern as a result of *m*-AMSA treatment was different from that seen after VP16 treatment (Pommier *et al.*, 1991). How this would affect the ability of thermotolerant cells (via elevated hsp levels?) to become resistant to VP16 but not *m*-AMSA is also as yet unclear. In any case, the observed resistance to VP16 was not accompanied by a reduced formation of cleavable complexes, as revealed by PFGE analysis (Figure 5). Li (1987) also observed no alterations in VM26-induced cleavable complex formation in thermotolerant cells. So neither drug accumulation nor drug-DNA interaction seems to be altered in the thermotolerant cells. This is also in accordance with hsp27-mediated protection against doxorubicin toxicity that was shown to be unrelated to reduced drug accumulation via a multidrug-like mechanism: immunoblot analysis revealed no P-gp 170 overexpression, and drug accumulation was unaltered by hsp27 overexpression (Huot *et al.*, 1991). So, as suggested by Li (1987), the observed protection (by hsp) is likely to be at a later stage, e.g. in DNA damage processing. In relation to this, it is noteworthy that inducers of hsp27 phosphorylation alone already can lead to protection against, for example, doxorubicin and VM26 (Huot *et al.*, 1992). Hsp27 may exert phosphorylation-activated functions linked with growth signalling pathways (Landry *et al.*, 1992), and as such elevated hsp27 expression and (de)phosphorylation may alter the cell cycle progression of the thermotolerant cells after drug treatment, allowing more time for DNA damage processing. The fact that growth arrest has been found to coincide with hsp27 phosphorylation and hsp27 levels (Spector *et al.*, 1992) could be in accordance with this suggestion. Alternatively, drug-induced apoptosis might be reduced in thermotolerant cells.

In conclusion, heating cells before topoisomerase II drug treatment protects against DNA break formation, leading to a reduced cytotoxicity of the drugs. In cancer therapy these treatments should therefore be used with caution. The data indicate that the hyperthermia treatment does reduce the ability of the drug-topoisomerase II complex to form the cleavable complexes related to drug toxicity. Protein aggregation induced by heat may explain such an effect. Elevated heat shock proteins may be related to the observed resistance of thermotolerant cells to VP16 (but not *m*-AMSA). This protective mechanism seems to occur at a stage *after* DNA-drug interaction.

**Abbreviations:** *m*-AMSA, 4′-(9′-acrydinylamino)methanesulphon-*m*-aniside; VP16, etoposide; VM26, tenoposide; MAR, matrix-associated region; hsp, heat shock protein; hsc, heat shock cognate; dsb, double-strand break; PFGE, pulsed-field gelelectrophoresis.

**Acknowledgements**
Part of this work was financed by the Dutch Cancer Society (Grant 89-09). I would like to acknowledge Annette van Assen, Gerard Buist and Eddy de Boer for their technical assistance with some of the experiments.

### References

BAKIC M, BERAN M, ANDERSSON BS, SILBERMAN L, ESTEY E AND ZWELLING LA. (1986). The production of topoisomerase II-mediated DNA cleavage in human cells predicts their susceptibility to 4′-(9′-acridinylamino) methanesulfon-*m*-aniside. *Biochem. Biophys. Res. Commun.*, **134**, 638-645.

BEREZNEY R. (1984). Organization and functions of the nuclear matrix. In *Chromosomal Nonhistone Proteins*, Hnilica LS. (ed.) pp. 119-231. CRC Press: Boca Raton, FL.

BLOCHER D AND KUNHI M. (1990). DNA double-strand break analysis by CHEF electrophoresis. *Int. J. Radiat. Biol.*, **58**, 23-34.

BLOCHER D, EINSPENNER M AND ZAJACKOWSKI J. (1989). CHEF electrophoresis, a sensitive technique for the determination of DNA double strand breaks. *Int. J. Radiat. Biol.*, **56**, 437-448.

CARMICHAEL J, DEGRAFF WG, GAZDAR AF, MINNA JD AND MITCHEL JB. (1987). Evaluation of a tetrazolium-based colorimetric assay: assessment of chemosensitivity testing. *Cancer Res.*, **47**, 936-942.

CIOCCA DR, FUQUA SAW, LOCK-LIM S, TOFT DO, WELCH WJ AND MCGUIRE WL. (1992). Response of human breast cancer cells to heat shock and chemotherapeutic drugs. *Cancer Res.*, **52**, 3648-3654.

COCKERILL PN AND GARRARD WT. (1986). Chromosomal loop anchorage of the kappa immunoglobulin gene occurs next to the enhancer in a region containing topoisomerase II sites. *Cell*, **44**, 273–282.

COVEY JM, KOHN KW, KERRIGAN D, TILGEN EJ AND POMMIER Y. (1988). Topoisomerase II-mediated DNA damage produced by 4′-(9′-acridinylamino) methanesulfon-*m*-aniside and related acridines in L1210 cell and isolated nuclei: relation to cytotoxicity. *Cancer Res.*, **48**, 860–865.

DARBY MK, HERRERA RE, VOSBERG P AND NORDHEIM A. (1986). DNA topoisomerase II cleaves at specific sites in the 5′ flanking region of c-fos proto-oncogenes in vitro. *EMBO J.*, **5**, 2257–2265.

ENGELHARDT R. (1987). Hyperthermia and drugs. In *Recent Results in Cancer Research: Hyperthermia and the Therapy of Malignant Tumors*, Streffer C. (ed.) pp. 136–203. Springler: Berlin.

GASSER SM AND LAEMMLI UK. (1986). The organization of chromatin loops: characterization of a scaffold attachment site. *EMBO J.*, **5**, 511–518.

GLISSON B, GUPTA R, SMALLWOOD-KENTRO S AND ROSS W. (1986). Characterization of acquired epipodophyllotoxin resistance in a Chinese hamster ovary cell line: loss of drug-stimulated DNA cleavage activity. *Cancer Res.*, **46**, 1934–1938.

HUOT J, ROY G, LAMBERT H, CHRETIEN P AND LANDRY J. (1991). Increased resistance after treatments with anticancer agents of Chinese hamster cells expressing the human $M_r$ 27 000 heat shock protein. *Cancer Res.*, **51**, 5245–5252.

HUOT J, ROY G, LAMBERT H AND LANDRY J. (1992). Co-induction of HSP27 phosphorylation and drug resistance in Chinese hamster cells. *Int. J. Oncol.*, **1**, 31–36.

JORRITSMA JBM AND KONINGS AWT. (1983). Inhibition of repair of radiation-induced strand breaks by hyperthermia and its relationship to cell survival after hyperthermia. *Int. J. Radiat. Biol.*, **43**, 505–516.

KAMPINGA HH, LUPPES JG AND KONINGS AWT. (1987). Heat-induced nuclear protein binding and its relation to thermal cytotoxicity. *Int. J. Hypertherm.*, **3**, 459–465.

KAMPINGA HH, WRIGHT WD, KONINGS AWT AND ROTI ROTI JL. (1988). The interaction of heat and radiation effecting the ability of nuclear DNA to undergo supercoiling changes. *Radiat. Res.*, **116**, 114–123.

KAMPINGA HH, VAN DER KRUK G AND KONINGS AWT. (1989*a*). Reduced DNA break formation and cytotoxicity of the topoisomerase II drug 4′-(9′-acridinylamino) methanesulfon-m-aniside when combined with hyperthermia in human and rodent cell lines. *Cancer Res.*, **49**, 1712–1717.

KAMPINGA HH, WRIGHT WD, KONINGS AWT AND ROTI ROTI JL. (1989*b*). Changes in the structure of nucleoids isolated from heat-shocked cells. *Int. J. Radiat. Biol.*, **59**, 369–382.

KAMPINGA HH, TURKEL-UYGUR N, ROTI ROTI JL AND KONINGS AWT. (1989*c*). The relationship of increased nuclear protein content induced by hyperthermia to killing of HeLa cells. *Radiat. Res.*, **117**, 511–522.

KAMPINGA HH. (1993). Thermotolerance in mammalian cells: protein denaturation and aggregation, and stress proteins. *J. Cell Sci.*, **104**, 11–17.

KONINGS AWT. (1987). Effects of heat and radiation on mammalian cells. *Radiat. Phys. Chem.*, **30**, 339–349.

LANDRY J, LAMBERT H, ZHOU M, LAVOIE JN, HICKEY E, WEBER LA AND ANDERSON CW. (1992). Human hsp27 is phosphorylated at serines 78 and 82 by heat shock and mitogen-activated kinases that recognize the same amino acid motif as S6 kinase II. *J. Biol. Chem.*, **267**, 794–803.

LASZLO A. (1992). The effects of hyperthermia on mammalian cell structure and function. *Cell Prolif.*, **25**, 59–87.

LI GC. (1987). Heat shock proteins role in thermotolerance, drug resistance, and relationship to DNA topoisomerases. *NCI Monogr.*, **4**, 99–103.

MIRKOVITCH J, MIRAULT M-E AND LAEMMLI UK. (1984). Organization of the higher order chromatin loop: specific attachment sites on the nuclear scaffold. *Cell*, **39**, 223–232.

MIZUMO S, AMAGAI M AND ISHIDA A. (1980). Synergistic cell killing by antitumor agents by hyperthermia in cultured cells. *Gann*, **71**, 471–478.

OESTERREICH S, WENG CN, QIU M, HILSENBECK SG, OSBORNE CK AND FUQUA SAW. (1993). The small heat shock protein hsp27 is correlated with growth and drug resistance in human breast cancer cell lines. *Cancer Res.*, **53**, 4443–4448.

POMMIER Y, CAPRANICO G, ORR A AND KOHN KW. (1991). Distribution of topoisomerase II cleavage sites in simian virus 40 DNA and effects of drugs. *J. Mol. Biol.*, **222**, 909–924.

RICE GC AND HAHN GM. (1987). Modulation of adriamycin transport by hyperthermia as measured by fluorescence-activated cell sorting. *Cancer Chem. Pharmacol.*, **20**, 183–187.

RIOU J-F, GABILLOT M, PHILIPPE M, SCHREVEL J AND RIOU G. (1986). Purification and characterization of *Plasmodium berghei* DNA topoisomerase I and II: drug action, inhibition of decatenation and relaxation, and stimulation of DNA cleavage. *Biochemistry*, **25**, 1471–1479.

ROSEMANN M, KANON B, KONINGS AWT AND KAMPINGA HH. (1993). An image analysis technique for the detection of radiation-induced DNA fragmentation after CHEF electrophoresis. *Int. J. Radiat. Biol.*, **64**, 245–249.

ROWE TC, CHEN GL, HSIANG Y AND LIU LF. (1986). DNA cleavage by antitumor acridines mediated by mammalian DNA topoisomerase II. *Cancer Res.*, **46**, 2021–2026.

SAKKERS RJ, BRUNSTING JF, FILON AR, KAMPINGA HH, KONINGS AWT AND MULLENDERS LHF. (1995). Anchoring of DNA loops at the nuclear matrix: disturbances of matrix structure and function by heat shock (submitted).

SPECTOR NL, SAMSON W, RYAN C, GRIBBEN J, URBA W, WELCH WJ AND NADLER LM. (1992). Growth arrest of human B lymphocytes is accompanied by induction of the low molecular weight mammalian heat shock protein (Hsp28). *J. Immunol.*, **148**, 1668–1673.

UDVARDY A, SCHEDL P, SANDER M AND HSIEH T. (1986). Topoisomerase II cleavage in chromatin. *J. Mol. Biol.*, **191**, 231–246.

WANG JC. (1985). DNA topoisomerases. *Ann. Rev. Biochem.*, **54**, 665–697.

WARTERS RL, BRIZGYS LM, SHARMA R AND ROTI ROTI JL. (1986). Heat shock (45°C) results in an increase of nuclear matrix protein mass in HeLa cells. *Int. J. Radiat. Biol.*, **50**, 253–268.

WARTERS RL AND BARROWS LR. (1994). Heat sensitivity of HeLa S3 cell DNA topoisomerase II. *J. Cell. Physiol.*, **159**, 468–474.